# Transcutaneous Vagus Nerve Stimulation (tVNS) and the Dynamics of Visual Bistable Perception

**DOI:** 10.3389/fnins.2019.00227

**Published:** 2019-03-08

**Authors:** Marius Keute, Lisa Boehrer, Philipp Ruhnau, Hans-Jochen Heinze, Tino Zaehle

**Affiliations:** ^1^Department of Neurology, Otto von Guericke University Magdeburg, Magdeburg, Germany; ^2^Center for Behavioral Brain Sciences, Magdeburg, Germany; ^3^Department of Behavioral Neurology, Leibniz Institute for Neurobiology, Magdeburg, Germany

**Keywords:** tVNS, transcutaneous vagus nerve stimulation, GABA, bistable perception, Necker cube

## Abstract

Transcutaneous vagus nerve stimulation (tVNS) is widely used for clinical applications, but its mechanism of action is poorly understood. One candidate pathway that might mediate the effects of tVNS is an increase in GABAergic neurotransmission. In this study, we investigated the effect of tVNS on visual bistable perception, which is highly coupled to GABA. Participants were 34 healthy young subjects. We used a static (Necker cube) and a dynamic (structure from motion) bistable perception task. Each subject underwent tVNS as well as sham (placebo) stimulation for ∼45 min. We analyze effects of tVNS on percept durations by means of Bayesian multilevel regression. We find no evidence for a modulation of bistable perception dynamics through tVNS in either task, but the analyses do not ultimately confirm the null hypothesis either. We discuss different possible implications of our finding and propose that GABAergic effects of tVNS should be further investigated using more direct measures of GABA concentration, and, more generally, that a better understanding of the mechanisms of action of vagus nerve stimulation is needed. Finally, we discuss limitations of our study design, data analysis, and conclusions.

## Introduction

Transcutaneous vagus nerve stimulation (tVNS) is a relatively new method of non-invasive neural stimulation (Ventureyra, 2000) that is mostly employed as an adjunct therapy for drug-refractory epilepsy, but may have therapeutic potential for a variety of conditions, such as depression (Sackeim et al., 2001), tinnitus (Lehtimäki et al., 2013), autism spectrum disorders (Jin and Kong, 2017), cerebral ischemia (Ay and Ay, 2014), and others. It has been introduced as an alternative to invasive vagus nerve stimulation (iVNS). Effects of iVNS on norepinephrine (NE), acetylcholine (ACh), and gamma-aminobutyric acid (GABA) neurotransmission, mediated through activations in the nucleus of the solitary tract and the locus coeruleus, have been shown consistently (Ben-Menachem et al., 1995; Marrosu et al., 2003; Follesa et al., 2007; Albert et al., 2009; Nichols et al., 2011), and data from fMRI investigations suggest that central nervous effects of tVNS are similar to the effects of iVNS (Dietrich et al., 2008; Kraus et al., 2013; Frangos and Komisaruk, 2017). Therefore, it is commonly assumed that tVNS increases levels of NE, GABA and ACh in the central nervous system (Steenbergen et al., 2015; Van Leusden et al., 2015; Beste et al., 2016; Colzato et al., 2018), even though direct neurobiological evidence is pending.

In a recent study (Keute et al., 2018), we demonstrated effects of tVNS on automatic motor inhibition, a process tightly coupled to GABA concentration in the motor cortex (Boy et al., 2010). Effects of tVNS on other processes associated to GABA have been found, such as cortical excitability (Capone et al., 2015), action cascading (Steenbergen et al., 2015), response inhibition (Beste et al., 2016), and divergent thinking (Colzato et al., 2018). To further corroborate the engagement of a GABAergic pathway through tVNS, we now examined effects of tVNS on the dynamics of bistable perception, which is highly correlated to GABA concentration in the visual cortex (Van Loon et al., 2013).

Bistable perception means switching between multiple perceptual interpretations of a constant sensory (e.g., visual) input (Blake and Logothetis, 2002). Ambiguous figures are a well-known example of visual stimuli resulting in bistable perception, but there are dynamic, binocular, and auditory examples of bistable perception as well (Pressnitzer and Hupé, 2006). Individuals differ with respect to bistable perception dynamics, and several covariates for interindividual variation have been identified, such as structural characteristics of the parietal cortex (Kanai et al., 2010) and genetic contributions (Miller et al., 2010; Shannon et al., 2011). The inhibition account of bistable perception states that it arises from reciprocal inhibition of different stimulus-selective neural populations in the visual cortex (Blake and Logothetis, 2002; Wang et al., 2013). Alternative accounts have been proposed that emphasize interactions between perceptual and cognitive processes rather than low-level perceptual inhibitions (Sterzer et al., 2009). In favor of the inhibition account, however, it has been found that GABA concentration in the visual cortex as measured by magnetic resonance spectroscopy is positively correlated with perceptual stability, i.e., the average timespan during which perceptual interpretation remains constant, in several visual bistable perception tasks. Furthermore, pharmacological increase of GABA_A_ activity through administration of lorazepam increased perceptual stability (Van Loon et al., 2013). Motivated by these findings, Van Leusden et al. (2015) proposed to study effects of tVNS on bistable perception in order to further establish the link between tVNS and GABA-associated behavioral and perceptual effects.

Besides this GABA-dependence of perceptual stability in visual bistable perception, other neurotransmitter systems have been found to be involved. Percept duration is positively correlated to pupil diameter at the time of perception switch (Einhäuser et al., 2008), which is a reliable marker of NE activity (Gilzenrat et al., 2010). Moreover, an influence of the dopamine (Schmack et al., 2013) and serotonin (Kondo et al., 2011) systems has been discussed.

In the present study we investigated tVNS effects on static as well as dynamic visual bistable perception. Given that tVNS is assumed to increase GABAergic transmission, we expected bistable perception to be stabilized, i.e., a prolonging of perception epochs between two switches.

## Materials and Methods

### Participants and Procedure

Participants were 34 healthy volunteers (20 female) between 18 and 33 years of age (mean: 23.1 ± 3.0). All participants were right-handed, free from current or past neurological or psychiatric diseases, were under no medication (except for oral contraceptives) and had normal or corrected-to-normal vision. Written informed consent was obtained from all participants prior to the experiment. They received money (€8/h) or course credit as a reimbursement for participation. The study was carried out in accordance with the declaration of Helsinki and approved by the local ethics committee.

**FIGURE 1:**
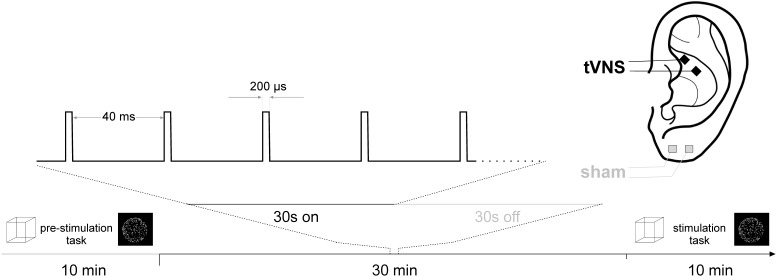
Experimental design.

Each participant underwent two experimental sessions, one involving active tVNS at the cymba conchae of the left ear and one involving sham (placebo) stimulation at the left ear lobe (cf. Figure 1). The order of tVNS and sham stimulation was randomized across participants. Both sessions were scheduled at the same daytime and at least 48 h apart at constant light conditions.

Each experimental session consisted of two experimental tasks, each run once before (pre) and once during stimulation (online). The order in which the two tasks were presented was randomized across sessions, but held constant within one session (i.e., between the pre and online run).

All stimuli were presented on a 24 inch screen at a vertical refresh rate of 60 Hz. Participants were seated at a distance of 70 cm to the screen. Responses were given by pressing the left and right control button on a PC keyboard. All experimental tasks were coded and run in MATLAB 2015^1^ using Psychtoolbox 3 ^2^.

In the static bistable perception task, a Necker cube (Kornmeier and Bach, 2005) was presented on the screen for 300 s. The cube consisted of black lines presented on a white background and subtended a visual angle of 7.0°. In this task, two spatial orientations of the cube can be perceived, in which either of the two central vertices can appear to be in front, i.e., closer to the observer. Participants were instructed to initially indicate whether they perceived the left or the right vertex to be closer by pressing the corresponding key and to indicate every switch in perceptual interpretation by pressing the key corresponding to the perception after the switch.

For the dynamic bistable perception task, referred to as structure from motion (SFM), a circular cloud of left- and right-moving dots was presented on the screen with a central fixation cross. These moving dots are perceived as an either left- or right-rotating sphere with the bistable perceptive interpretation being the direction of rotation (left vs. right) (Van Loon et al., 2013). Again, participants were asked to indicate the perceived direction of rotation initially and after every perceptual switch by pressing the associated key. The two dot clouds moved at an angular velocity of 23°/s around the vertical axis. The individual dot size was 6.6 arcmin in width and height. All dots were equal in luminance (white) on a gray background. The dot clouds covered a circular area with a diameter of 15.6° visual angle. After an initial presentation of a fixation cross, the task was presented for 300 s.

After the first run of the two tasks, electrical stimulation started and was administered for 30 min prior to the second run of the tasks to give stimulation effects time to unfold. Stimulation continued throughout the online run of the tasks (cf. Figure 1).

For stimulation, medical Ag/AgCl electrodes (Ambu Neuroline ^3^), cut to a size of 4 mm × 4 mm and mounted on a piece of silicone at a center-to-center distance of 1 cm were used. Electrical conductance between the electrode and the skin was established using a small amount of Genuine Grass adhesive electrode cream (Natus Neurology ^4^). For tVNS, the electrodes were placed in the cymba conchae of the left ear, for sham stimulation at the left earlobe. Across conditions and participants, the anode was placed more rostral. Stimulation pulses were generated by a medical stimulation device (Digitimer DS7 ^5^) at a current intensity of 3 mA and a pulse width of 200 μs, triggered by an Arduino Uno circuit board ^6^ programmed to a stimulation cycle of 30 s stimulation at 25 Hz, followed by a 30 s break.

### Data Analysis

We analyzed percept durations (PD), which were computed as the time difference between two reported switches. When the same key was pressed multiple subsequent times, only the first press was counted, such that all PD values describe the time span between two changes in perception. The time before the first and after the last keypress was excluded from the analysis. Furthermore, PDs shorter than 200 ms were considered lapses and excluded from further analysis. We excluded subjects if they had carried out two keypresse or less, i.e., no percept switches, in at least one of the four runs of a task. Furthermore, we excluded subjects if the time between their first and last keypress was shorter than 150 s, i.e., if less than half of the runtime was available for analysis, in at least one of the four runs of a task. We computed mean PDs for each subject in each run. Data from 29 subjects for the Necker cube task and from 25 subjects for the SFM task entered the final analysis.

We analyzed mean PDs by means of Bayesian multilevel regression using the *brms* library in R and Stan (Bürkner, 2017). We constructed a linear model of PD with time (pre- vs. post-stimulation), stimulation (sham vs. tVNS), and time × stimulation interaction as fixed effects. As random effects, we specified subject-wise random intercepts to account for repeated measures. We used weakly regularizing Gaussian priors (μ = 0, σ = 15) for the model coefficients of all three fixed effects (McElreath, 2016). Posterior distributions of the parameters have been obtained by Markov chain Monte Carlo (MCMC) sampling in Stan (Gelman et al., 2015) with 5000 iterations per chain, the first 2000 iterations being discarded as “warm-up” iterations, and four independent sampling chains. Since our effect of interest was the time × stimulation interaction, we compared the model with interaction to a model without it using Bayes factors. Moreover, we report the posterior distribution of the interaction model coefficient as estimated in the 12000 iterations of the MCMC procedure, alongside the 95% highest density interval (HDI), i.e., the 2.5 and 97.5% percentiles of the posterior effect size distribution.

## Results

### Necker Cube

**FIGURE 2:**
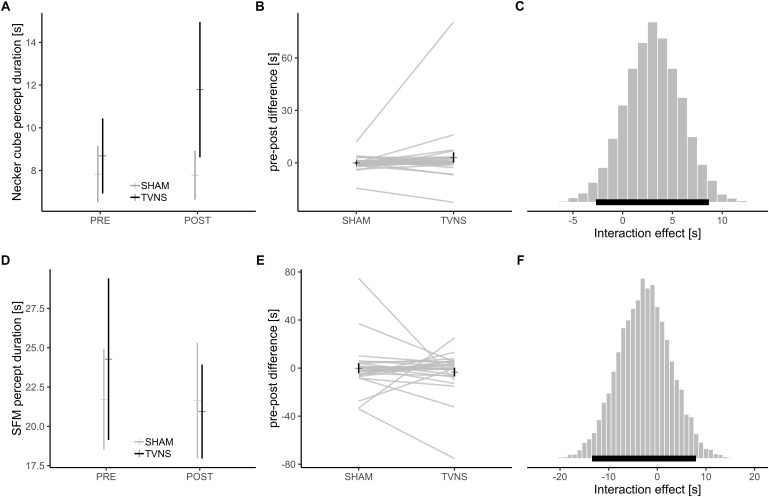
**(A)** Mean ± SEM of percept durations in the Necker cube task; **(B)** Mean ± SEM of pre-online change in percept durations in the Necker cube task, gray lines: individual subjects; **(C)** Posterior distribution of time × stimulation interaction in the Necker cube from the Bayesian multilevel model, black bar: 95% highest density interval of interaction effect; **(D–F)** Equivalents for the SFM task.

Mean overall PD for the static bistable perception task (Necker cube) was 9.0 s. The Bayesian sampling procedure estimated a mean time × stimulation interaction of 3.0 s. The 95% HDI was -2.7<*b*<8.7 (Figure 2A–C). Bayes factor model comparison favored the model without interaction over the model with interaction (BF: 2.9).

### Structure From Motion

Mean overall PD for the dynamic bistable perception task (SFM) was 22.1 s. The Bayesian sampling procedure estimated a time × stimulation interaction of -2.6 s. The 95% HDI was -13.4<*b*<7.9 (Figure 2D–F). Bayes factor model comparison favored the model without interaction over the model with interaction (BF: 2.5).

### Correlations Between Tasks

Collapsed over all task runs, PDs were moderately correlated (*ρ* = 0.42, *p* < 0.001) between both tasks. Spearman’s *ρ* is reported because percept durations in both tasks differed significantly from the normal distribution (both *p* < 0.005 in Lilliefors–Kolmogorov–Smirnov tests).

## Discussion

In this study we asked whether tVNS affects the dynamics of visual bistable perception. As suggested previously, an increase of GABAergic activity through tVNS should result in slower dynamics of visual bistable perception (Van Leusden et al., 2015). We analyzed percept durations using Bayesian multilevel regression analyses. Bayesian analyses, other than most inferential statistical methods, allow in principle to accept the null hypothesis, based on the quantiles of the posterior distributions or Bayes factors (Kruschke, 2013). In our analyses, the 95% HDI of coefficient distributions in both analyses included zero, but both intervals were rather wide. Moreover, Bayes factors favored a model without interaction effect for both experimental tasks, but the magnitude of both Bayes factors was rather low (<3). In sum, we find evidence for a null effect of tVNS on the dynamics of visual bistable perception, which is, however, not fully conclusive. On the other hand, no tendency toward a non-zero effect size is apparent from either experimental task, so we tentatively accept the null hypothesis. The moderately high correlations between the two tasks indicate that they capture similar processes underlying bistable perception (cf. Carter and Pettigrew, 2003).

Even though our results are not fully conclusive, there are several possible interpretations of our results. First, tVNS might have a different effect on GABA transmission in different parts of the brain. Even though there is no *a priori* reason to assume that GABAergic effects of tVNS are different between the motor and visual cortex, the results from our recent study (Keute et al., 2018), alongside another study investigating the effects of tVNS on cortical excitability (Capone et al., 2015) indicate that effects of tVNS on GABA transmission might have a more complex spatial distribution in the brain than just a whole-brain increase, but a systematic investigation of this is pending. Therefore, we cannot rule out that tVNS affects GABA transmission in the motor but not in the visual cortex. We suggest that the spatial distribution of GABAergic effects of tVNS should be investigated using more direct measures such as magnetic resonance spectroscopy. Moreover, it seems to be an oversimplification of the mechanism of action of tVNS if hypotheses about its behavioral or physiological effects are derived simply based on increases of NE, ACh, and GABA. Further central and peripheral candidate pathways of both tVNS and iVNS have been found, including serotonergic (Dorr and Debonnel, 2006; Grimonprez et al., 2015), plasticity-promoting (Biggio et al., 2009; Borland et al., 2016), anti-inflammatory (Ottani et al., 2009; Kaczmarczyk et al., 2018), and peripheral autonomic (Clancy et al., 2014) mechanisms. An integrative model of these mechanisms and their interaction is pending.

Second, despite the robust correlation (Van Loon et al., 2013), GABA in the visual cortex is not the only neurotransmitter system with an influence on visual bistable perception. Other neurotransmitters, such as dopamine (Schmack et al., 2013) and norepinephrine (Einhäuser et al., 2008; Hupé et al., 2009) are potential mediators of visual bistable perception dynamics. Norepinephrine is considered an important target neurotransmitter of tVNS (Badran et al., 2018). Even though a tVNS-induced increase in norepinephrine transmission should have a stabilizing effect on bistable perception (Einhäuser et al., 2008), i.e., should have the same direction as a tVNS induced increase in GABA transmission, interactions between neurotransmitter systems may be more complex. Moreover, bistable perception dynamics underlie numerous inter- and intraindividual variations, such as gender, personality traits, practice (Scocchia et al., 2014), genetic differences (Miller et al., 2010; Shannon et al., 2011; Schmack et al., 2013), or clinical conditions (Vierck et al., 2013).

Third, there are limitations to our experimental design. Several participants had to be excluded based on the criteria described above, which might indicate that the parameters of our experimental paradigm have not been optimally tuned. Longer stimulus presentations and improved control of visual attention, e.g., by using a chin-rest, might improve the overall data quality. However, given our data, there is no apparent reason to assume that this would have led to the discovery of a tVNS effect.

In sum, we do not find any positive evidence for a tVNS effect on visual bistable perception, but our data remain inconclusive inasmuch as they do not ultimately confirm the null hypothesis either. We did not find evidence for a simple link between tVNS, GABA transmission and stabilized bistable perception.

## Ethics Statement

The study was approved by the ethics committee of the Medical Faculty, Otto von Guericke University Magdeburg and is in accordance with the Declaration of Helsinki.

## Author Contributions

MK and TZ conceived and designed the experiments. LB performed the experiments. MK, LB, PR, and TZ analyzed the data. MK, PR, and TZ wrote the article. H-JH contributed to materials and analysis tools.

## Conflict of Interest Statement

The authors declare that the research was conducted in the absence of any commercial or financial relationships that could be construed as a potential conflict of interest.

## Footnotes

1 www.mathworks.com

2 www.psychtoolbox.org

3 www.ambu.com

4 www.natus.com

5 www.digitimer.com

6 www.arduino.cc
